# Quality of Cancer Care in Tanzania as Experienced by Patients: A Qualitative Study

**DOI:** 10.1177/23779608231157332

**Published:** 2023-02-16

**Authors:** Paulo L. Kidayi, Hélio Manhica, Christina C. Mtuya, Mahande Michael Johnson, Serventi Furaha, Ragnhild E. Aune, Gunilla Björling

**Affiliations:** 1 Faculty of Nursing, 108094 Kilimanjaro Christian Medical University College, Moshi, Tanzania; 2 Department of Global Public Health, 27106 Karolinska Institutet, Stockholm, Sweden; 3 25548 Sophiahemmet University, Stockholm, Sweden; 4 Institute of Public Health, 108094 Kilimanjaro Christian Medical University College, Moshi, Tanzania; 5 Cancer Care Centre, Kilimanjaro Medical Centre, Moshi, Tanzania; 6 Department of Material Sciences, 8018 Norwegian University of Technology and Science, Trondheim, Norway; 7 Department of Neurobiology, Care Sciences and Society, 27106 Karolinska Institutet, Stockholm, Sweden; 8 Department of Nursing, School of Health and Welfare, 4161 Jönköping University, Jönköping, Sweden

**Keywords:** oncology, qualitative research, cancer, advance practice nurses, oncology care

## Abstract

**Introduction:**

Cancer is a disease of public importance in Tanzania. Considering a limited health care system with few cancer centers and low health literacy in general, people are diagnosed at late stage and face difficulties in accessing care for their cancer. All these challenges affect the caring situation for the nurses who meet the patients at the cancer care centers.

**Objective:**

This study aimed to explore the journey of cancer care experienced by patients with cancer.

Research questions:

How do patients experience the quality of care at the cancer care centers?

How do patients experience the family’s and the community's role?

**Methods:**

Semi-structured qualitative interviews were carried out with 15 patients treated for colorectal-, breast-, or prostate cancer in three cancer care centers in Tanzania. A purposive sampling was used. Qualitative content analysis according to Graneheim and Lundman was employed.

**Results:**

Three main themes and six sub-themes emerged. The main themes were *experiences of cancer care services, the role of the family, community challenges and cancer care.* The sub-themes were *communication, resource allocation at the cancer care centers, fmily's denial, family challenges, limitations of primary care and misconceptions,* and *accessibility of cancer service and the health care system.* The findings show that patients experienced incapacitated health care facilities for cancer care, misconceptions in the community and challenges for the families.

**Conclusions:**

The quality of cancer care in Tanzania needs to be improved, patients face challenges in all levels of health care facilities, including families, and the community at large. The distance to oncology care, economic hardship, and lack of knowledge in the community including families, lead to late diagnosis and suffering for the patients. There is a great need for education regarding cancer within healthcare, as well as in the community, to change the situation for patients with cancer.

## Introduction

Cancer is an emerging public disaster globally. The incidence was 19.3 million; and the mortality rate was 10 million in 2020, with a high fatality rate in Africa of 7.2% (Sung et al., 2021). For low and middle-income countries there are huge challenges in supporting patients with cancer as cancer centers are few, and lack of both human and non-human resources is evident (MoHCDGEC, 2018; WHO, 2017). In Tanzania in 2020, a total of 40,464 new cancer cases and 26,945 deaths were reported (Sung et al., 2021). Few cancer centers and low health literacy in general lead to late diagnosis (MoHCDGEC, 2018, 2021; Rick et al., 2021). Lack of healthcare insurance among patients, and oncology nursing training (The United Republic of Tanzania, Ministry of Health and Social Welfare, 2013) might affect the quality of care and the caring situation for the nurses who meet the patients at the cancer care centers. Patients’ perception of the quality of care they receive are essential for providing patient-centered care and services and for increasing patients' involvement in the care (WHO, 2017). In the present study, we are interested in studying cancer patients’ experiences of the cancer care in cancer care centers, in the community, as well as in the family.

## Review of Literature

Cancer care centers in Tanzania are few and urban-based (Kohi et al., 2019), situated in consultants’ hospitals and none at primary health care facilities and there is a lack of nursing oncology training. Therefore, cancer patients are likely to receive care from nurses trained in general nursing (The United Republic of Tanzania, Ministry of Health and Social Welfare, 2013). Distance to cancer care centers and poverty also limit early diagnosis and treatment, the majority reach late stages 3 and 4 (MoHCDGEC, 2018; Akuoko et al., 2017; Rick et al., 2021). These challenges the nurses in providing person-cantered care for the patients. Studies have critically analyzed the care given in hospital settings (Banaser et al., 2017; Kohi et al., 2019), however, they did not capture the whole experience of patients from the time of suspecting cancer, seeking care at health care facilities and undergo treatment, and how they experienced the quality of care. Caring for cancer patients is challenging for nurses, with late diagnosis, lack of resources and few cancer centers (Piot et al., 2016).

There is scarce research regarding quality of cancer care in Sub Saharan Africa, but a study from Ethiopia showed that lack of knowledge and clinical skills among nurses acted as a barrier for nurses to deliver quality care to these patients (Getachew et al., 2020). Research from three European countries by Charalambous et al. (2016) shows that perceived nursing care quality and perceived individuality in care are directly associated with trust in nurses. In addition, these factors are affected by health status. In a study on quality of care perceived by cancer patients’, it was shown that health status was the only factor associated with cancer patients’ assessments of care quality attributes. Cancer itself was the strongest determinant of the care quality perceptions (Suhonen et al., 2018). However, there is a lack of knowledge on how patients with cancer experience the cancer care in Tanzania. To improve the quality of care for these patients and the situation for the nurses working in the cancer care centers, it is important to explore this topic. The present study will derive information regarding the quality of cancer care utilizing a holistic approach to examine the journey of cancer care, experienced by patients. Based on the results of the study, future interventions can be developed to improve oncology care in healthcare facilities and in the community. The purpose of the study was, therefore, to explore the journey of cancer care services in Tanzania as experienced by patients.

## Methods

### Design

The study has a descriptive qualitative explorative inductive design using semi-structured interviews, which aimed to explore how cancer patients in Tanzania experience their cancer journey. The chosen method was considered appropriate as a qualitative inductive explorative approach that aims to capture the lived experience of persons in a certain context in alignment with Polit and Beck (2017). The study method coincides with Granheim and Lundman (2004) in analyses and writing qualitative research. The study is reported according to the COnsolidated criteria for REporting Qualitative studies, COREQ-checklist, which is used for reporting qualitative data developed by Tong et al. (2007) (see Supplemental Appendix 1).

### Research Questions

How do patients experience the quality of care at the cancer care centers?

How do patients experience the family’s and the community's role?

### Sample

The study was carried out in the three major cancer care centers in Tanzania. These major cancer hospitals are the only centers that provide radiation, chemotherapy, and surgery in the country, and were, therefore, selected for screening of study participants. The participants were chosen based on high prevalence of types of cancer from a national perspective. We used a purposive sampling to retrieve the participants (Polit & Beck, 2017). The interviewer (first author) screened the study participants. In total, *n* = 15 informants were included in the study and saturation point was reached at the 13th participant. The last two participants were interviewed to confirm that no new information could be obtained beyond the number of study participants. To use a purposive sampling method was appropriate to capture the target population (Polit & Beck, 2017).

### Inclusion Criteria and Exclusion Criteria

Adults >18 years of age, diagnosed with breast-, colorectal-, or prostate cancer since  > 6 months who gave their voluntary written informed consent were included. Terminally ill patients were excluded, as well as those who did not want to participate in the study.

### Ethical Consideration

Ethical clearance certificate was obtained from the National Institute for Medical Research in Tanzania. The Declaration of Helsinki (World Medical Association, 2013) was followed throughout the entire course of the study. The participants were not in a position of dependence in relation to the researchers. Prior to the interviews, the participants received written and verbal information about the study and were informed of their right to withdraw without any explanation. The participants provided written informed consent.

### Data Collection Method

Data was collected through semi-structured qualitative interviews during May and June 2021. The semi-structured interview guide was developed by the researchers and back and forward translation of the interview guide (English to Swahili and vice versa) was done by two independent experts and pre-tested to ensure the validity of the guide before being used (Polit & Beck, 2017). The interview guide is displayed in Supplemental Appendix 2. Two pilot interviews were performed to test and adjust the guide. However, there was no need for adjustment of the interview guide, and it remained the same during the entire data collection which ascertained the topics covered in all interviews. The interviewer strived to be open to the stories told by the participants so that no critical experiences were missed (Graneheim & Lundman, 2004). The interviews took place in a quiet room convenient for the patients at the cancer care centers and lasted from 40 min to 1 h. Each interview was recorded, with the permission of the participant, and field notes were taken during the interviews. Before participation, all study informants gave their written informed consent (Polit & Beck, 2017).

### Data Analysis

Content analysis was employed by using the principles of Graneheim and Lundman (2004) to identify prominent themes and patterns in the text. First, the interviews were transcribed verbatim, and then the text was read through several times by three of the researchers, with vast knowledge of qualitative methods, independently, to reach a deeper understanding of the transcribed text. Thereafter, the researchers analyzed the text by breaking down data into smaller units, coding, and naming the units according to the content in the text. The units were then grouped into themes based on shared concepts and discussed among the three researchers. Three main themes and seven sub-themes emerged from the analyzed text and agreement was reached. Those themes were then discussed in the large research group. Four criteria were applied to establish trustworthiness: credibility, dependability, transferability, and confirmability, which are vigorously recommended by Lincoln and Guba (1985) for qualitative research; to strengthen the findings of the study (Polit & Beck, 2017) and avoid researchers’ interpretation of data during analysis.

## Results

The study explores how patients, attending specialized cancer care centers in Tanzania, perceive and experience the cancer care, including the role of the family and the community.

### Sample Characteristics

The informants were patients, diagnosed since a minimum of 6 months, with breast-, prostate-, or colorectal cancer. In total, 15 patients were included, in the present study, whereof 6 men and 9 women. Their age ranged from 40 and 88 years and the mean age was 61.5 years. The majority were married, had a primary level of education, a salary, and lived in urban areas. Individual demographic data of the informants are presented in Table 1. In Table 2, demographic data are presented on group level.

**Table 1.** Participants’ Individual Socio-Demographic Characteristics, *n* = 15.

Respondent	Age (sex)	Type of cancer	Type of care	Patients’ awareness of cancer stage	Types of treatment received	Time since diagnosis	Metastasis	Education level	Marital status	Occupation	Residence
Patient 1	81(M)	Prostate	Outpatient	Not informed	Chemotherapy/Surgery	2 years	Yes	Primary	Married	Peasant	Rural
Patient 2	52 (F)	Breast	Inpatient	IV	Chemotherapy	2.7 years	Yes	Secondary	Married	Self-Employed	Urban
Patient 3	88 (M)	Prostate	Outpatient	III	Chemotherapy/Radiation	3 years	Yes	Primary	Married	Retired officer	Rural
Patient 4	49 (F)	Breast	Outpatient	Early stage	Chemotherapy/Surgery	1.11 years	Yes	Secondary	Married	Self-employed	Urban
Patient 5	62 (F)	Colorectal	Outpatient	Not informed	Chemotherapy/Surgery	13 years	Yes	Primary	Married	Retired officer	Urban
Patient 6	47 (F)	Breast	Outpatient	II	Surgery/chemotherapy	1 year	No	Primary	Married	Business	Urban
Patient 7	48 (F)	Breast	Outpatient	I	Chemotherapy	2.7 years	No	University	Married	Teacher	Rural
Patient 8	81 (M)	Prostate	Outpatient	Not informed	Chemotherapy	3 years	No	Secondary	Married	Retired	Urban
Patient 9	66 (M)	Prostate	Outpatient	Not informed	Surgery/Chemotherapy	5 years	No	Primary	Married	Retired	Rural
Patient 10	50 (F)	Breast	Inpatient	Not informed	Surgery/Chemotherapy	2.7 years	Yes	Primary	Single	Small business	Urban
Patient 11	60 (F)	Breast	Inpatient	Not informed	Surgery/Chemotherapy	1.7 years	Yes	None	Divorced	Peasant/business	Rural
Patient 12	42 (F)	Colorectal	Inpatient	Not informed	Surgery/Chemotherapy/Radiation	1.7 years	Yes	University	Married	Teacher/business	Urban
Patient 13	40 (F)	Colorectal	Outpatient	Not informed	Surgery	1.7 years	Not informed	Primary	Married	Self-employed	Urban
Patient 14	85 (M)	Prostate	Inpatient	Not informed	Surgery/Radiation/Chemotherapy	6 years	No	Primary	Married	Retired	Urban
Patient 15	71 (M)	Prostate	Outpatient	Not informed	Chemotherapy/Radiation	1 year	Yes	University	Married	Retired	Urban

**Table 2.** Demographics of the Study Participants *n* = 15, Group Level.

Variable	*N* (%)	Mean (SD)	Range
*Age*			
Years		61.5 (14.9)	42–81
*Time since diagnosis* Years		3.2 (3)	1–13
*Sex*			
Male	6 (40)		
Female	9 (60)		
*Type of cancer*			
Breast cancer	6 (40)		
Colorectal cancer	3 (20)		
Prostate cancer	6 (40)		
*Metastatic disease*			
Yes	9 (60)		
* *No	5 (33.3)		
* *Not informed	1 (6.7)		
*Type of care*			
In-patient care	5 (33.3)		
Out-patient care	10 (66.7)		
*Type of treatment*			
Surgery/chemotherapy	7 (46.7)		
Chemotherapy	3 (20)		
Chemotherapy/radiation	2 (13.3)		
Surgery/chemotherapy/ radiation	2 (13.3)		
Surgery	1 (6.7)		
*Level of education*			
* *Primary school	8 (53.3)		
* *Secondary school	3 (20)		
* *University	3 (20)		
* *None	1 (6.7)		
*Occupation*			
* *Business	2 (13.3)		
* *Self-employed	3 (20)		
* *Teacher	2 (13.3)		
Peasant	2 (13.3)		
Retired	6 (40)		
*Marital status*			
Married	13 (86.7)		
Single/divorced	2 (13.3)		
*Residence*			
Rural	5 (33.3)		
* *Urban	10 (67.7)		

### Research Questions Results

**Figure 1.**
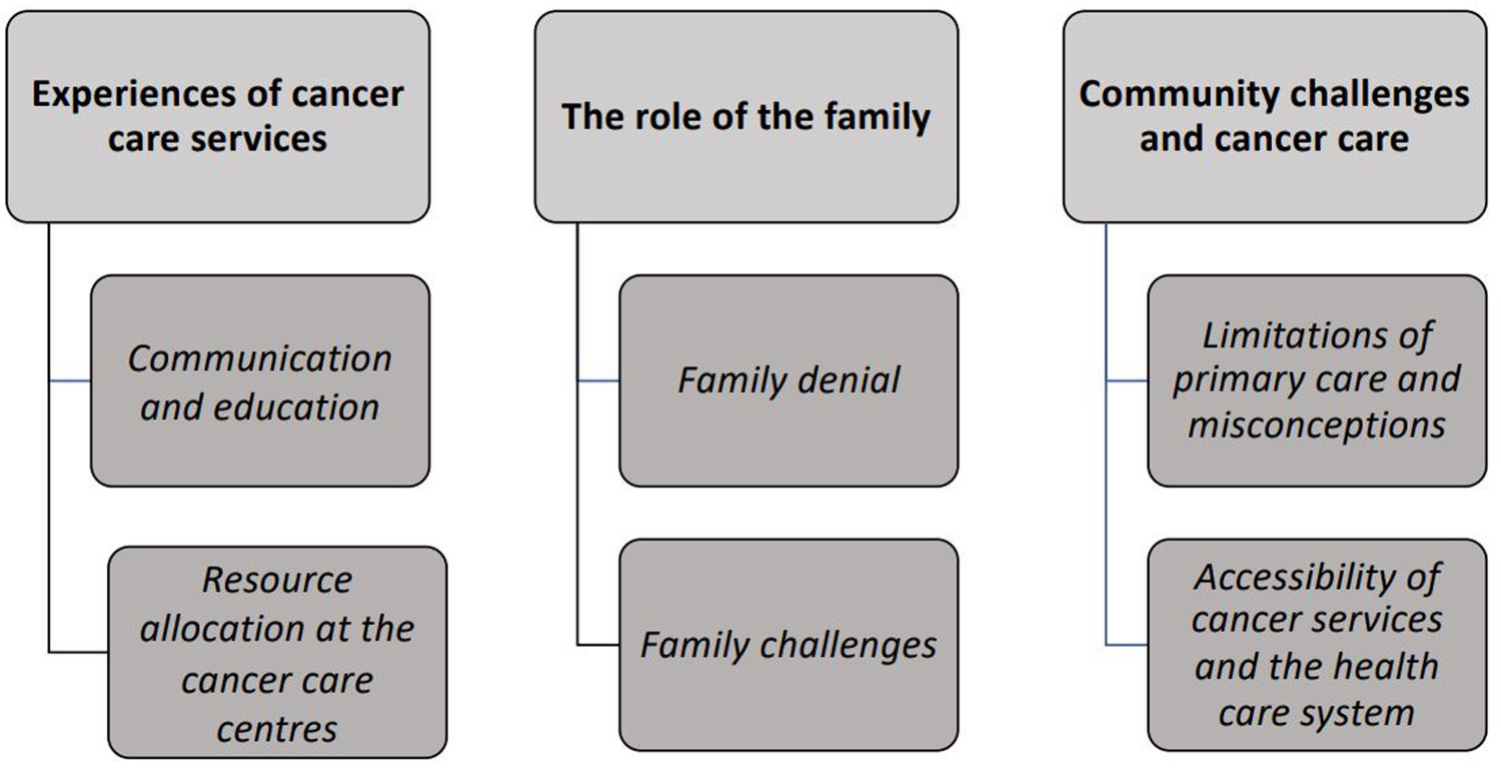
Composed themes and sub-themes.

A content analysis approach according to Graneheim and Lundman (2004) was used, and three main themes and six subthemes were identified as shown in Figure 1. The main themes were *Experiences of cancer care services, The role of the family,* and *Community challenges and cancer care.*

#### Main Theme: Experiences of Cancer Care Services

This theme refers to patients’ opinions on the service provided in specialized cancer hospitals. It includes *Communication and education* and *Resource allocation at the cancer care centers.* The experiences of the patients vary as they describe being treated well and badly by the nurses. The care services in the hospital often include very long waiting for different investigations and treatments. The participants emphasized the importance of education about the disease and treatment for both patients and healthcare workers to improve the care situation. Regarding the quality of facility in the cancer care centers, there was a shortage of equipment and materials which affected the patients.

##### Sub-Theme: Communication and Education

The informants narrated having both good and bad communication with the healthcare staff at the cancer care centers. Some informants experienced that the caring situation was good and they felt that the healthcare staff paid them attention. Some informants described that, after diagnosis, the healthcare staff clarified the steps of the treatment and its effect on the patient, for example, advantages and side effects. The patients, thereafter, made an independent decision on treatment.

…. The service is good, the physician and nurse explained to me very well, I am going to start this treatment, then I will continue. So, they did the operation, after recovery I was referred to the cancer care centre to see the physician. I didn’t go home, and I got a good explanation at the cancer care centre and was satisfied, then started chemotherapy… (Study participant P6)

On the other hand, some informants experienced both language barriers and incivility from the healthcare staff. All informants acknowledged that there was a communication barrier between health care staff and the patients’ receiving services at cancer care centers, that is, regarding time for investigations, retrieving medicine, at the laboratory etc. The patients were not given any instructions and feedback on the process until they asked why they, for example, had to wait for so long time in that section. They patients were forced to find the information themselves which was frustrating.

… In the area of communication, it is a problem…You stay until you ask the nurse how far? …… They tell you not yet, wait. (Study participant P4)

…Here customer care doesn’t exist! Especially for patients seeking treatment for the first time. No-one is responsible to direct people…, when you ask, they always reply, -Just go over there, and they don’t explain any further. …Here customer care is nothing. If I calculate, 90%, they do not have customer care. Nobody, not even to direct you to the laboratory. (Study participant P7)

In addition, some informants described that some of the nurses were rude and used harsh language when the patients were talking to them. The informants also expressed that some nurses were inexperienced and performed malpractice which could hurt patients and jeopardize patient safety. The patients stressed that it is important that all nurses should be trained on how to handle cancer patients in cancer care centers and thereby avoid malpractice. One informant narrated that the nurses need more knowledge in oncology nursing as they encounter the patients unprofessionally without sympathy and knowledge.

 …I know because of the weight of the work even these junior nurses they should attend seminar… for example, one nurse put chemotherapy in a wrong vein and I am medical personnel; when I told her, - why are you doing that, she said” – Don’t you teach me how to work” and that drip did not run and I got severe pain like burn and I shouted and removed my hand… (Study participant P5)

. I, personally, I think nurses should be educated on how to give services to patients with cancer…They are supposed to meet them with sympathy. (Study participants P14)

##### Sub-Theme: Resource Allocation at the Cancer Care Centers

All informants narrated that the cancer care centers were congested with patients. For example, the rooms for the chemotherapy section are very small, and males and females share the same room. Sometimes patients were given a prescription to collect drugs at the pharmacy but whenever they reached there, there were no drugs, and they were advised to purchase them in private pharmacies.

….. Once the drugs are prescribed, it takes time until you get that drug. Or sometimes, they tell you there is no drug. For me it happened twice, they told me there is no drug…. (Study participant P7)

Many informants described that the cancer care centers face shortages of investigation equipment, and sometimes the informants were directed to perform the investigations out of the cancer care centers, which rendered in high costs for the patients. Moreover, health care providers also failed to give service because some materials or equipment were missing in the hospitals.

… Sometimes there is no equipment! …and this means that they tell us to go to another place to do investigations; …Health care providers are not empowered because sometimes they might lack specific materials or instruments and thus fail to provide some services. (Study participant P10)

Most of the informants narrated, that the cancer care centers had shortage of staff which contributed to long waiting hours for the patients. The patient could have arrived very early at the center for chemotherapy, but was called very late to receive the medication, which rendered unnecessary living costs in the hospital due to the delay of the services.

…They give good services; but they are very few…., we spend a long time in waiting for the oncologist, and even when we go to the laboratory, we are waiting for a long time…. (Study participant P9)

#### Main Theme: The Role of the Family

This theme describes how the family affect the cancer patient. The subthemes that emerged in this theme are *family denial* and *family challenges.* Being diagnosed with cancer does not only affect the patient, but the whole family. Several aspects were emphasized by the informants, that is, cost for treatment, support from family members, loss of income, lack of knowledge. All these factors affect the person that is sick.

##### Sub-Theme: Family Denial

Many informants narrated that when being diagnosed with cancer, their families deny them to get services in the cancer centers. They think cancer is incurable and, therefore, it will not be necessary to attend health care service. Most informants also described that the family decided where to get the cancer treatment, and sometimes they were told to attend traditional healers, though it did not work. Instead, the condition became worse, and eventually they chose to go back to the cancer centers.

They checked… I had cancer, the family said, there is alternative medicine somewhere. You will be cured…because the breast had a little tumour…. So, I started that treatment(traditional), but I found the condition was going in the wrong direction! Then I met one nurse who advised me to go to the hospital. (Study participant 6)

First, people, they believe that cancer is incurable…. Once the recurrence occurs, the patient will die. Even me, before coming to the hospital, my family told me to go for alternative medicine. (Study participant P10)

##### Sub-Theme: Family Challenges

All informants acknowledged the role of the family in supporting them at home and when attending the cancer care centers. One of the most challenging issues, described by all informants, was economic hardship. The informants described that the living costs at home, payment to the cancer clinic, and the fact that the distance to the clinics are too far, were heavy burdens. Therefore, they tried herbal or traditional medicine.

…Because in other health care facilities, when they mention the cost, you ask yourself, -Am I going to manage? The investigation itself is too expensive. You ask yourself, when you start cancer treatment, -Am I going to manage? …. People, they choose after what they can manage, thus they choose herbal medicine. (Study participant P2)

#### Main Theme: Community Challenges and Cancer Care

This theme refers to community defies of cancer care. The two sub-themes are *Limitations of primary care and misconceptions* and *Accessibility of cancer services and the health care system.* There is scarce access to health care facilities in the community where the patient can be diagnosed and get treatment. The traditional beliefs are very strong, the knowledge about cancer is low, and the costs are high even for those covered by insurances. All these obstacles lead to late diagnosis and poor prognosis.

##### Sub-Theme: Limitations of Primary Care and Misconceptions

Many informants narrated that they attend health care facilities when suspecting having a tumor. Due to incapacitated primary health care facilities on cancer care, there was a delay of referral from the district or regional hospital to cancer care centers, rendering a delay in diagnosis and treatment.

…Ah… in primary health care facilities. there is nothing! There are not enough specialists; no investigations, especially in the villages. (Study participant P14)

Furthermore, the misconception of cancer in the community is devastating. There are rumors, and misbeliefs which affect adherence to cancer treatment and many patients do not go to the hospital or they fear to go to the hospital. People in the community think that a person diagnosed with cancer will die and the cancer patient is neglected.

People neglects cancer patients, they see them as dead, … Even if relatives, they have money, they don’t like to send them to the hospital. They say hospital is for death!… They think cancer is an uncurable disease. The government should take this as a challenge, as there so many cancer patients. and some they are convinced to go to traditional healers. Whenever they go there, there is no cure. So, the government should educate the community. (Study participant P11)

In addition, the informants narrated that people in the community sometimes even do not like to hear your progress nor receive patients’ telephone calls.

Community members they are ready to escape from hearing your status. Even if you call, they don’t answer. They think you want to ask for money. (Study participant 10)

Many informants acknowledged the need for health education in the community to create awareness on disease and treatment to improve cancer services and survival. The informants expressed that education within the community was necessary, as well as in hospitals.

Firstly, provide education in district hospitals and villages because there are so many people who die; some without knowing they have cancer. (Study participant P11)

However, some informants narrated they are valued and assigned responsibilities in the community. For example, one informant narrated health care providers need to provide patient education, so they can share their knowledge in the community, or healthcare providers can come and teach community members about cancer.

One day I was asked to teach in the church concerning cancer, but I was afraid, and I said what am I going to teach, as I have not enough knowledge? If possible, health care workers, they could come in the village and give health education on cancer. (Study participant P5)

##### Sub-Theme: Accessibility of Cancer Service and Health Care System

This sub-theme refers to patients’ experiences regarding availability, affordability and even distribution of cancer care services, as well as follow up and provision of health education, both in rural and urban areas.

Informants narrated that the services are urban based, with high cost which few community members manage to attend. Community misconceptions and traditional beliefs limit the patients to reach the cancer centers. A healthcare system that does not cover for medical treatment for all citizens also contributes to suffering.

.…I am telling you, there in the villages in the rural areas, there are so many patients… but they don’t come because they can’t afford those costs… If they would announce the service is free, you will see many patients coming… (Study participants P5)

The means required to reach cancer care center is a big challenge, as there are only three cancer care centers in Tanzania. This is a challenge and due to long distance to the cancer care centers patients fail to get treatment. It was also stated by the participants that there is a need for cancer treatment centers in the district and regional hospitals to increase the availability of treatment to patients. This could assist much, as patients are travelling far distances to consultants and national cancer care centers.

… Tell me, I come from one region, I sell maize, getting transport fair and cancer treatment are expensive, even if the family can contribute. They might contribute only for the first and the second time. Next time they leave you alone…It would be better if each region, or preferably, they could refer you to your region to get those chemotherapy cycle. -Yeah, it would be very nice instead of attending cancer hospitals! This could reduce cost for transport, as treatment is not done in one day. Today, they make investigation, tomorrow you get the results and next day you get chemotherapy, then you wait to know, whether you will be able to travel back home or not. (Study participant P7)

…Regarding treatment with chemotherapy, let it be offered at district and regional hospitals so we can get it in our own region and thereby avoid unnecessary cost!. (Study participant P11)

The informants narrated that there are no clear linkages and follow-up of the patients when they are at home, neither while on treatment, nor after being discharged. None of the health care providers inform them about available services in the community and they are not referred if there should be any.

## Discussion

The aim of the study was to explore the journey of cancer services in Tanzania as experienced by patients. Three themes emerged, *Patients’ experiences of cancer care services, Experiences of support and care from the family,* as well as *Experiences of community challenges and cancer care.* The results disclose the importance of a patient cantered care with a caring perspective from the caregivers, as described by Bergbom et al. (2021).

### Experiences of Cancer Care Services

The informants expressed they experienced a good caring relationship with the oncologists and nurses which motivated the patients to comply with treatment and promoted follow-up. However, the patients found the cancer care centers to be short of staff and knowledge among healthcare staff. Some of the nurses had harsh language and used rude expressions to the patients. This is in contrary with research from Banaser et al. (2017), where there were positive nurse–patient interactions. Good nurse–patient relationship promotes patient-centered care and facilitates patients to make their informed decision and appropriate choice thus adhere to treatment (Imaizumi et al., 2021). The informants in the present study narrated having a bad experience of the caring environment at the cancer care center. The environment on the oncology department has shown to have impact on the patients’ wellbeing (Edvardsson et al., 2006).

Furthermore, incapacitated quality of health care facilities in the present study is the sign of unmet cancer services both in urban and rural residents. They were facing limited space and inadequate non-human resources. For example, some equipment for diagnostic and performing procedures and prescribed drugs are missing. These results are supported by findings from previous studies in Tanzania and Zimbabwe (Tapera et al., 2019; Yohana et al., 2011). Patients purchase drugs in a private pharmacy at a high cost and sometimes fail to get them; or wait until they are available at cancer care centers. This forces patients to discontinue treatment and accelerates lost to follow-up and recurrence. Moreover, Suija et al. (2013) found there were overcrowded patients at cancer centers. This demands the government to improve the infrastructure to encourage cancer patients to attend cancer care. There is a shortage of staff, including an oncologist, leading to long waiting hours. Also, inadequate knowledge and skills on cancer disease and treatment among nurses which risk patients; demanding political will for capacity building in nursing oncology for nurses. In contrary to other studies (Kvåle & Bondevik, 2010), nurses were competent and patients feel safe and protected (Imaizumi et al., 2021). It is important that patients and staff develop a caring relationship to enhance the experience of treatment for the patient.

### The Role of the Family

There are challenges for the families, that is, cost for treatments and loss of income. These challenges lead to late diagnosis, as the patient and family try traditional medicine before medical treatment due to high costs. However, many of the participants appreciated the support from their families, which are in line with findings from previous studies (Ogunkorode et al., 2021; Cinar et al., 2018) where the support from families promoted patients’ coping mechanisms with their disease and treatment. Nonetheless, Arora et al. (2007) found that the support drops within a year from diagnosis. Furthermore, the level of support could be due to cultural context, as high cost and low income is challenging for the family. The present study shows that caring for cancer patients is demanding both for patients living at home and at cancer care centers. Some families also make the decision for patients to receive cancer treatment, which overpowers the patient's autonomy. This is also confirmed by a study from Saud Arabia (Buetto & Zago, 2015), where the families overpowered the patients’ decisions to receive treatment. According to Bergbom et al. (2021), the caring relationship between the carer and the patient is essential. The families are included in the caring team of the patient, and it is important to enhance knowledge to improve the caring experience of the patient.

### Community Challenges and Cancer Care

The findings show that there is a lack of knowledge in the society about cancer and the primary care has no cancer services, patients are diagnosed late and misbelieves are common. These results are confirmed by a study from Ethiopia, where it was found that a lack of cancer services in primary health care facilities act as barriers for early cancer detection (Getachew et al., 2020). Patients experienced challenges on complying with chemotherapy despite of received education at cancer centers due for instance to long distances or high costs. Especially in remote areas, the distance acts as a barrier to accessing cancer services. These findings are confirmed by previous studies (Mahapatra et al., 2016; Marcusson-Rababi et al., 2019; Rick et al., 2021). Furthermore, the patients in the present study experienced that belief and misconception that cancer is incurable, and that a person with cancer is being bewitched, are common among some community members, which hinder patients both with or without money to attend cancer services.

The findings from this study show a low health literacy among patients, families, community members and health care providers, and are in line with other studies (Rick et al., 2021; Suija et al., 2013; Tapera et al., 2019). Low health literacy among people in general is common and affects the time of diagnosis and the caring relationship. In the present study, none of the patients were linked for continuum cancer care services in the community; even for minor illnesses whenever attended at primary health care facilities they were referred to cancer care centers. This has consequences for patients with low health literacy and for those facing economic hardship, as they cannot afford to go to the cancer care center choose a traditional healer or alternative medicine instead. The findings are in line with a study by Mahapatra et al. (2016) where patients did not get services at the primary care centers. On the other hand, previous studies by Suija et al. (2013) and Marcusson-Rababi et al. (2019) show that patients can get help from community volunteers at home and in the community for cancer care after discharge from cancer hospitals and are offered great support which enhances a good caring relationship.

## Strength and Limitations

The study had an independent approach to examine the whole process patients undergo from the period suspecting tumor until being diagnosed with cancer. Patients face challenges as individuals, in the family, in the community, and within healthcare. However, by only interviewing patients, the study missed information from administrative authorities to address barriers and facilitating factors on the provision of quality care to cancer patients, which could be a limitation. However, the study did not include patients who rejected or dropped out from cancer treatment, which might have influenced the results. Inclusion of dropouts from cancer treatment could have added alternative explanations on the cancer services existing in the country including families and communities. The participants’ duration of disease since diagnosis ranged from 1 to 6 years, which could have had an implication on the recall bias. On the other hand, they were still followed up at the cancer care centers.

The study's trustworthiness can be considered good as the information derived from non-dependable participants and the settings where the study conducted consisted of different national and consultant hospitals. Data was collected from multiple settings in one national cancer care center and two zonal referral cancer care centers with a diversified population of different cancer to ensure its credibility. Therefore, the findings are transferable to the other settings particularly in low- and middle-income countries in Sub-Saharan Africa as other LIC also face similar challenges. The informants were selected based on the high prevalence of types of cancer in the country, which reflects the national and international arena as a means of dependability. Furthermore, the researchers who performed the interviews followed the interview guide and had no previous relation to the informants. The confirmability was achieved as the researcher collected data independently and one independent individual performed data transcriptions and translations. Furthermore, three independent researchers with vast knowledge of qualitative methods analyzed the data and come up with varied interpretations, and later agreed on the themes and sub-themes to be the final findings. This enhanced the transparency and obtaining valid findings from this study in accordance with Graneheim and Lundman (2004) and Lincoln and Guba (1985).

## Implications for Practice

The findings from this study show that, capacity building for nurses, and health education to the community and families’ members are critical to reduce misconception of disease and cancer treatment, and thus improve low health literacy, facilitate access to cancer care services, and improve the situation for patients with cancer. Therefore, it is important to include the family in the care of cancer patient in clinical practice.

## Conclusions

The quality of cancer care in Tanzania needs to be improved. The study revealed that patients are facing huge challenges during their cancer journey, both at hospital-, family-, and community levels. The quality of cancer care needs to be improved. Incapacitated health care facilities, traditional beliefs, and misconceptions in families and the community act as barriers to providing quality cancer care services. It is important to keep in mind that caring for this patient group affects not only the patient but the family, the cancer care team, staff in the community and all contribute to the caring culture.

## Supplemental Material

sj-docx-1-son-10.1177_23779608231157332 - Supplemental material for Quality of Cancer Care in Tanzania as Experienced by Patients: A Qualitative Study Click here for additional data file. Supplemental material, sj-docx-1-son-10.1177_23779608231157332 for Quality of Cancer Care in Tanzania as Experienced by Patients: A Qualitative Study by Paulo L. Kidayi, Hélio Manhica, Christina C. Mtuya and Mahande Michael Johnson, Serventi Furaha, Ragnhild E. Aune, Gunilla Björling in SAGE Open Nursing

sj-docx-2-son-10.1177_23779608231157332 - Supplemental material for Quality of Cancer Care in Tanzania as Experienced by Patients: A Qualitative Study Click here for additional data file. Supplemental material, sj-docx-2-son-10.1177_23779608231157332 for Quality of Cancer Care in Tanzania as Experienced by Patients: A Qualitative Study by Paulo L. Kidayi, Hélio Manhica, Christina C. Mtuya and Mahande Michael Johnson, Serventi Furaha, Ragnhild E. Aune, Gunilla Björling in SAGE Open Nursing
