# Supplementary material for: Quality of Cancer Care in Tanzania as Experienced by Patients: A Qualitative Study
Source: SAGE Open Nurs. 2023 Feb 16;9:23779608231157332. doi: 10.1177/23779608231157332 (PMC9940171; doi:10.1177/23779608231157332)
Supplement: sj-docx-1-son-10.1177_23779608231157332 - Supplemental material for Quality of Cancer Care in Tanzania as Experienced by Patients: A Qualitative Study [file sj-docx-1-son-10.1177_23779608231157332.docx]

**Appendix 1. Consolidated criteria for reporting qualitative studies (COREQ): 32-item checklist Tong et al 2007: Quality of cancer care in Tanzania as experienced by patients - A qualitative study**

| SNO |  |  |  |
| --- | --- | --- | --- |
|  | **Domain 1: Research team and reflexivity** |  |  |
|  | **Personal Characteristics** |  |  |
| 1 | Interviewer/facilitator | Which author/s conducted the interview? | The first author |
| 2 | Credentials | What were the researcher’s credentials? | PhD student, Registered Nurse (RN), MSc Epidemiology and Biostatistics |
| 3 | Occupation | What was their occupation at the time of the study? | Assistant Lecturer |
| 4 | Gender | Was the researcher male or female? | Male |
| 5 | Experience and training | What experience or training did the researcher have? | Trained both in qualitative and quantitative research |
|  | **Relationship with participants** |  |  |
| 6 | Relationship established | Was a relationship established prior to study commencement? | No |
| 7 | Participant knowledge of the interviewer | What did the participants know about the researcher? e.g., personal goals, reasons for doing the research | Participants informed the purpose, reasons for doing research |
| 8 | Interviewer characteristics | What characteristics were reported about the interviewer/facilitator? e.g. Bias, assumptions, reasons and interests in the research topic | None |
|  |  |  |  |
|  | **Domain 2: study design** |  |  |
|  | **Theoretical framework** |  |  |
| 9 | Methodological orientation and Theory | What methodological orientation was stated to underpin the study? e.g. grounded theory, discourse analysis, ethnography, phenomenology, content analysis | Content analysis; Graneheim and Lundman (2004) |
|  | **Participant selection** |  |  |
| 10 | Sampling | How were participants selected? e.g. purposive, convenience, consecutive, snowball | Purposive |
| 11 | Method of approach | How were participants approached? e.g. face-to-face, telephone, mail, email | Face to Face |
| 12 | Sample size | How many participants were in the study? | Fifteen (15) |
| 13 | Non-participation | How many people refused to participate or dropped out? Reasons? | None |
|  | **Setting** |  |  |
| 14 | Setting of data collection | Where was the data collected? e.g. home, clinic, workplace | Clinic |
| 15 | Presence of non-participants | Was anyone else present besides the participants and researchers? | No |
| 16 | Description of sample | What are the important characteristics of the sample? e.g. demographic data, date | Demographic data |
|  | **Data collection** |  |  |
| 17 | Interview guide | Were questions, prompts, guides provided by the authors? Was it pilot tested? | Yes, pilot tested |
| 18 | Repeat interviews | Were repeat interviews carried out? If yes, how many? | No |
| 19 | Audio/visual recording | Did the research use audio or visual recording to collect the data? | The research use audio recording to collect the data |
| 20 | Field notes | Were field notes made during and/or after the interview? | Field notes made during and/or after the interview |
| 21 | Duration | What was the duration of the interviews? | 45 minutes to 1 hour |
| 22 | Data saturation | Was data saturation discussed? | Yes |
| 23 | Transcripts returned | Were transcripts returned to participants for comment and/or correction? | No |
|  | **Domain 3: analysis and findings** |  |  |
|  | Data analysis |  |  |
| 24 | Number of data coders | How many data coders coded the data? | Three (3) |
| 25 | Description of the coding tree | Did authors provide a description of the coding tree? | Yes |
| 26 | Derivation of themes | Were themes identified in advance or derived from the data? | Derived from the data |
| 27 | Software | What software, if applicable, was used to manage the data? | None |
| 28 | Participant checking | Did participants provide feedback on the findings? | No |
|  | Reporting |  |  |
| 29 | Quotations presented | Were participant quotations presented to illustrate the themes / findings? Was each quotation identified? e.g. participant number | Yes |
| 30 | Data and findings consistent | Was there consistency between the data presented and the findings? | Yes |
| 31 | Clarity of major themes | Were major themes clearly presented in the findings? | Yes |
| 32 | Clarity of minor themes | Is there a description of diverse cases or discussion of minor themes? | Yes |

Establish
