# Supplementary material for: Quality of Cancer Care in Tanzania as Experienced by Patients: A Qualitative Study
Source: SAGE Open Nurs. 2023 Feb 16;9:23779608231157332. doi: 10.1177/23779608231157332 (PMC9940171; doi:10.1177/23779608231157332)
Supplement: sj-docx-2-son-10.1177_23779608231157332 - Supplemental material for Quality of Cancer Care in Tanzania as Experienced by Patients: A Qualitative Study [file sj-docx-2-son-10.1177_23779608231157332.docx]

**Appendix 2.** Semi-structured interview guide to explore quality of Care among cancer patients attending specialized hospitals providing oncology services in Tanzania.

| SNO | Question |
| --- | --- |
|  | Sociodemographic/clinical characteristics   1. Sociodemographic characteristics   Age------------Gender---------------Educational level-----------------Marital status---------  Occupation-------------Address: District---------------- Residence: urban vs rural--------   1. Clinical Characteristics   Stage diagnosed--------------Type of treatment received/pattern-------------------------  Duration on treatment:----------------------------Post treatment-------------------  Year since diagnosis------------------------Cancer recurrence/metastasis--------------------- |
| 2 | Health care facility care |
|  | 1. Where did you start to seek cancer services and why?   (Probe more on referral procedure if any?)  Primary health care facilities (name)  Regional hospital  Tertiary hospital  Specialized cancer Centre (name) |
|  | 1. How do you find the services at the oncology hospitals?   Explore more on: Accessibility & affordability, infrastructure, capacity, service available (diagnostic & treatment), Distance, Availability of supply   1. What is your perception on the treatment cost at oncology hospital? |
| 3 | Health care providers perspective from the patient view |
|  | 1. What were the challenges to start cancer treatment (chemotherapy, surgery, radiotherapy and hormonal therapy at oncology hospital? 2. How did the nurses and other staff help you to cope with disease and treatment burden? 3. How do you perceive the communication with the health care staff?   Probe (In & Outpatient care, scheduled appointment, equal treatment, waiting hours (time to service), communication, stigma and discrimination)   1. How do staffing and material for caring patients, such as diagnostic, treatment modalities, drugs affect the service and care? 2. What are the best and worst things in the care provided from health care providers? What are your opinion on reasons and improvements? |
| 4 | Treatment compliance and prognosis |
|  | 1. How did the prescribed treatment disturbed adherence to reduce disease burden? 2. What impact has the health care providers on your treatment compliance? |
| 5 | Services and follow-up from cancer hospitals for patients after discharge to home |
|  | 1. How do you perceive patients’ linkages to available services at the PHCF, palliative care, and community for psychosocial & economic support after being discharged from oncology hospital? 2. How do health care providers make follow up to continuity of care? 3. How often do you attend the clinic at the oncology hospital and what are the challenges? |
|  | 1. What are the misconceptions of the disease and treatment, and how does it affect the care, provided at oncology hospitals? |
|  | 1. What are the challenges that health care facilities face in giving high quality cancer care at the clinic and at home to the patients? |
| 6 | Family and community support and role |
|  | 1. Is there any social support in the community and what are their role in supporting cancer patients? |
|  | 1. Which impact have distance to hospital, travelling, living and home care cost on patients with cancer? Misconceptions? Traditional believes etc.? |
| 7 | Health service improvement? |
|  | 1. What are your views in improving cancer services in Tanzania?   At hospital, home, link to services in community, palliative care |
